# Stromal fibroblast activation protein alpha promotes gastric cancer progression via epithelial-mesenchymal transition through Wnt/ β-catenin pathway

**DOI:** 10.1186/s12885-018-5035-9

**Published:** 2018-11-12

**Authors:** Jiuyang Liu, Chaoqun Huang, Chunwei Peng, Fei Xu, Yan Li, Yonemura Yutaka, Bin Xiong, Xiaojun Yang

**Affiliations:** 1 Department of Gastrointestinal Surgery, Zhongnan Hospital of Wuhan University, No. 169 Donghu Road, Wuchang District, Wuhan, China; 2 Hubei Key Laboratory of Tumor Biological Behaviors & Hubei Cancer Clinical Study Center, Wuhan, 430071 China; 3 Department of Thyroid and Breast Surgery, Zhongnan Hospital of Wuhan University, Wuhan, China; 4 Department of General Surgery, Yingshan Renmin Hospital, Yingshan, 438700 China; 5 Department of Peritoneal Cancer Surgery, Beijing Shijitan Hospital, Capital Medical University, Beijing, 100038 China; 6 Peritoneal Dissemination Center, Kishiwada Tokushukai Hospital, Kishiwada, 596-0032 Japan; 7 Department of Surgery, Kusatsu General Hospital, Shiga, 600-8189 Japan

**Keywords:** Gastric cancer, Peritoneal metastasis, Fibroblast activation protein alpha, Epithelial-mesenchymal transition

## Abstract

**Background:**

To investigate the influence of fibroblast activation protein alpha (FAP) derived from cancer-associated fibroblasts (CAFs), as well as potential mechanism of epithelial mesenchymal transition (EMT), on gastric cancer (GC) progression.

**Methods:**

Correlation between CAFs-derived FAP and clinical results has been studied by using 60 GC cases. To confirm this relationship, SGC7901 cells were co-cultured with pre-established FAP-overexpressed fibroblasts in vitro and the characteristics including proliferation, migration, invasion and apoptosis abilities were detected subsequently. Meanwhile, SGC and GES1 cells cocultured with FAP-overexpressed fibroblasts were treated with cis-platinum for apoptotic analysis. The underlying EMT was detected by analyzing expression level of E-cadherin, ZO-1, N-cadherin, Vimentin, α-SMA, DKK1 and LEF-1 through western blot and immunofluorescence staining assay. Finally, the tumor-promoting ability of FAP was investigated by utlizing a xenograft gastric cancer nude mouse model.

**Results:**

It show that FAP has a high-risk correlation with the malignant level of clinical outcomes in GC patients. FAP promotes the ability of proliferation, migration, invasion, apoptosis-inhibition of SGC7901 cells and induces apoptosis of GES1 cells in vitro. The mechanism study shows that epithelial markers have been down-regulated and mesenchymal markers and Wnt/β-catenin signal pathway related proteins have been up-regulated. Animal assay suggests that tumor burden has been enhanced by FAP significantly in vivo.

**Conclusions:**

Stromal FAP could be a potential prognostic biomarker in GC by promoting cancer progression via EMT through Wnt/ β-catenin signal pathway.

**Electronic supplementary material:**

The online version of this article (10.1186/s12885-018-5035-9) contains supplementary material, which is available to authorized users.

## Background

Gastric cancer (GC) remains the fourth most common cancer and the fifth leading cause of cancer-related mortality worldwide [1, 2]. The postoperative invasion and metastasis have long been the lethal causes of death and great challenges for GC patients even after multimodality clinical treatments [3]. And almost 60% of all causes of GC death is due to peritoneal carcinomatosis (PC) [4]. According to recent new insights, PC was regarded as a regional tumor progression majorly occurred in abdomen pelvic cavities [5, 6].

The underlying mechanisms of GC PC has been a worldwide research hotspot, and more efforts were focused on the dynamic and complex PC progression. Momentum evidence has indicated that tumor microenvironment (TME) plays a crucial role in cancer progression [7, 8]. The co-evolution of cancer cells and stromal functional cells or molecules constitutes significant hallmarks of cancer [9]. Cancer associated fibroblasts (CAFs) act as key orchestrators in TME by directly protecting cancer cells from host immune attacks, and promoting cancer progression by complex mechanisms, for instance epithelial-mesenchymal transition (EMT) [10, 11]. Whether EMT could partly explain the cross talk between GC cells and stromal CAFs required further studies [12].

Fibroblast activation protein alpha (FAP), a homodimeric integral membrane gelatinase of the serine protease family, is selectively expressed by CAFs in stromal compartment [13, 14]. FAP could exerte profound influence on clinical outcomes of several human malignancies. For instance, FAP overexpression correlated with suppressed lymphocyte-dependent immune reactions and poor survival of non-small cell lung cancer and pancreatic adenocarcinoma [15, 16]. However, stromal FAP derived from CAFs in GC remained to be confirmed, as well as the regulatory mechanisms [17].

In this study, we have conducted experiments in vitro and in vivo to further characterize the biological processes associated with stromal FAP overexpression in GC. Based on the pre-established FAP-overexpressed fibroblasts (HELF^FAP^), the proliferation, invasion, migration, as well as anti-apoptosis abilities of SGC7901 cells in co-cultured model were investigated. Moreover, correlations between FAP and Wnt/β-catenin pathway was also detected to ascertain the potential role of EMT during GC progression. Taken together, we described the tumor promoting functions of stromal FAP, which might account for GC progression.

## Materials and methods

### Patients and follow-up

There were 60 GC cases included in this study, all of which have received radical operation at the Department of Gastrointestinal Surgery, Zhongnan Hospital of Wuhan University (Wuhan, China) from February 2009 to April 2011. Major clinicopathological characteristics including age, gender, tumor diameter, and TNM stages were collected. In addition, the information of follow up was available. TNM stages were determined according to the UICC/AJCC 7th TNM staging system of GC. The primary endpoint for this study was overall survival (OS), which was defined as the interval from the date of surgery to GC related death. Written informed consent was obtained from the patients with the study protocol approved by the ethics committee of Zhongnan Hospital of Wuhan University. The study was undertaken in accordance with the ethical standards of the World Medical Association Declaration of Helsinki.

### Immunohistochemistry staining

Routine IHC method was performed for the staining of FAP. The primary antibody was rabbit anti-human monoclonal antibody against FAP (ab227703, Abcam, UK, dilution 1/200), with corresponding horseradish peroxidase (HRP) conjugated secondary antibody (ab6721, Abcam, UK, dilution 1/200). The FAP positive CAFs were indicated by both morphological features and the IHC reaction results. The reaction products were visualized with diaminobenzidine (DAB, DAKO, Denmark). Then the slides were evaluated by two senior pathologists, who were blinded to the patients’ clinical features and outcomes. A consensus was achieved using a multi-headed microscope in case of discrepancy. In brief, at least 4 standard-compliant vision fields of FAP expression (magnification, × 200) per patient was considered to be adequate, with no focus on hotspots. The digital images were captured under Olympus BX51 fluorescence microscope equipped with Olympus DP72 camera (Olympus Optical Co., Ltd., Tokyo, Japan). Identical settings were used for every photograph, so as to minimize the selection bias.

### Cell culture

The SGC7901 cell line (human gastric cancer cell lines), GES1 cell line (normal mucosal epithelium cells), and HELF cell line (human embryonic lung fibroblasts; Cat NO.: CL-0281) were cultured in Dulbecco’s modified Eagle’s medium (DMEM) supplemented with 10% Fetal Bovine Serum (FBS), 100 IU/ml penicillin and 100 mg/ml streptomycin in a humidified atmosphere with 5% CO_2_ at 37 °C.

### Construction of HELF^FAP^ cells with overexpression of FAP

The lentivirus FAP-copGFP (1 × 10^8^ TU/ml) and a negative control (NC) were purchased from GenePharma (Shanghai, China). HELF cells seeded in six-well plates were transfected with control or lentivirus FAP-copGFP according to the manufacturer’s instructions. The multiplicity of infection (MOI) in this study was 50:1. Then puromycin was used to establish the stable transfected HELF cell line (HELF^FAP^). SGC7901 co-cultured with HELF^FAP^ and HELF^NC^ cells were used for further experiments.

### CCK8 assay

Cholecystokinin-8 (CCK-8) assay (Dojindo, Japan) was performed to detect the cell viability and cell growth. Briefly, 6000 viable gastric cancer cells were seeded in 96-well plates. After specific treatment, each well was mixed with 10 μL CCK-8 and incubated for additional 1 h. The OD values were detected at an absorbance of 450 nm.

### Colony formation assay

A colony formation assay was used to detect cells survival. For clonogenicity analysis, 1000 viable co-cultured SGC7901 cells were placed in six-well plates. Culture medium was changed every two days. After two weeks of incubation, colonies were fixed with 4% paraformaldehyde and stained with crystal violet. The cells were photographed and the numbers of colonies were scored.

### Wound healing assay

SGC7901 cells seeded in 6-well plates were scratched, washed with PBS supplemented with 1% FBS and treated as indicated. The cells were photographed by phase contrast microscope at 24 h in several pre-marked spots. Then the mean distance between both edges of cell free area was calculated.

### Transwell migration and invasion assays

The polycarbonate membrane in the transwell chambers were precoated with Matrigel with 1:40 dilution (Corning, USA) in 37 °C and air dried. There were 15,000 cells seeded and adhered in each chamber. After 24 h, the cells were fixed with 4% paraformaldehyde (PFA) and stained by 0.1% crystal violet, the number of migrated cells were counted and statistically analyzed. For migration assay, no Matrix gel was required.

### Flow cytometry

SGC7901 cells were placed in 12-well plates overnight, and then treated with compounds according to the manufacturer. Cells were then harvested, washed twice with pre-cold PBS, and evaluated for apoptosis by double staining with FITC-conjugated annexin V and propidium iodide (PI) (MultiSciences, Hangzhou, China) for 30 min in the dark. To assess the cell cycle, harvested cells were labeled with PI (5 mg/ml) in the presence of binding buffer (MultiSciences, Hangzhou, China) in darkness for 30 min.

### Real-time RT-PCR

Total RNA was extracted using RNA simple Total RNA kit (TIANGEN, Beijing, China). cDNA was generated with a first-strand cDNA synthesis Kit (Thermo, Waltham, MA) using the protocol recommended by the manufacturer.

The one-step real-time quantitative PCR were carried out in a 20 μl reaction mixture containing 10 μl 2 × SYBR Premix EX Taq II (Takara, Tokyo, Japan), 0.4 μM primers, and 1 μl of template cDNA. The primers were listed in Additional file 1: Table S1. All real-time RT-PCRs were performed at CFX96 real-time PCR detection system (Bio-Rad, Hercules, CA). Fluorescence was measured at the end of the annealing period of each cycle to monitor amplification. Glyceraldehyde-3-phosphate dehydrogenase (GAPDH) was used as internal reference.

### Western blotting

Cells were washed with cold PBS twice and prepared in RIPA lysis buffer, and western blot analysis was performed as described previously [18]. Specific primary antibodies used were the following: DKK1 (ab61275, Abcam); LEF1 (ab217378, Abcam); ZO-1 (61–7300, Thermo); Vimentin (ab92547, Abcam); N-cadherin (ab76011, Abcam); E-cadherin (ab1416, Abcam). Anti-GAPDH was purchased from Aspen (Wuhan, China). After incubating with a fluorescein-conjugated secondary antibody (Li-Cor, Lincoln, NE, USA), the membranes were analyzed using an Odyssey fluorescence scanner (Li-Cor, Lincoln, NE, USA).

### Immunofluorescence staining (IF)

SGC7901 cells were seeded on 24 mm coverslips, fixed with 4% PFA for 30 min, treated by 0.1% Triton X-100 and blocked in 5% BSA for 1 h at room temperature. Sequentially the fixed cells were incubated with primary antibody at 4 °C overnight (E-cadherin, ab1416, Abcam, dilution 1/50; α-SMA, ab32575, Abcam, dilution 1/300), washed with PBS and incubated with Cy3-labelled or FITC-labelled secondary antibody for 1 h at room temperature. The nuclei were labelled with DAPI (2 mg/ml), and the immunofluorescence staining was analyzed using a fluorescence microscope (Olympus BX5, Olympus Optical Co., Ltd., Tokyo, Japan).

### In vivo xenograft assay

Six-week-old female BALB/cA nu/nu mice were purchased from Vital River Laboratory Animal Technology Company (Beijing, China) and maintained in an Animal Biosafety Level 3 Laboratory at the Animal Experimental Center of Wuhan University. All animal experiments were performed according to the Wuhan University Animal Care Facility and National Institutes of Health guidelines. Approximately 3 × 10^6^ SGC7901 cells and 1 × 10^6^ HELF^FAP^ cells (Group I, *n* = 5), 3 × 10^6^ SGC7901 cells and 1 × 10^6^ HELF^NC^ cells (Group II, n = 5) were harvested and suspended in 200 ml of PBS and Matrigel (BD Bio-science, USA) (1:1) and injected subcutaneously into the right flank of each mouse. The size of subcutaneous tumors was recorded every two days. Five weeks later, mice were sacrificed, and the tumors were removed. The weight of tumors was recorded and statistically analyzed. The xenograft tumor slides were incubated with the following primary antibodies: anti-CD31 was purchased from ABclonal (Boston, USA) and anti-Ki67 from Cell Signaling Technology (Boston, USA). Anti-rabbit or anti-mouse peroxidaseconjugated secondary antibody (ABclonal, Boston, USA) and diaminobenzidine colorimetric reagent solution (Dako, Carpinteria, CA) were used. The staining processes were performed according to standard methods.

### Statistical analysis

All experiments were performed at least three times. Data are presented as the mean ± SD. All statistical analyses were performed using GraphPad Prism 6.0 (GraphPad, San Diego, CA). One-way ANOVA and Student’s t-test were applied to determine statistical significance. A value of two-sided *P* < 0.05 was considered statistically significant.

## Results

### The clinical significance of stromal FAP in GC

**Fig. 1:**
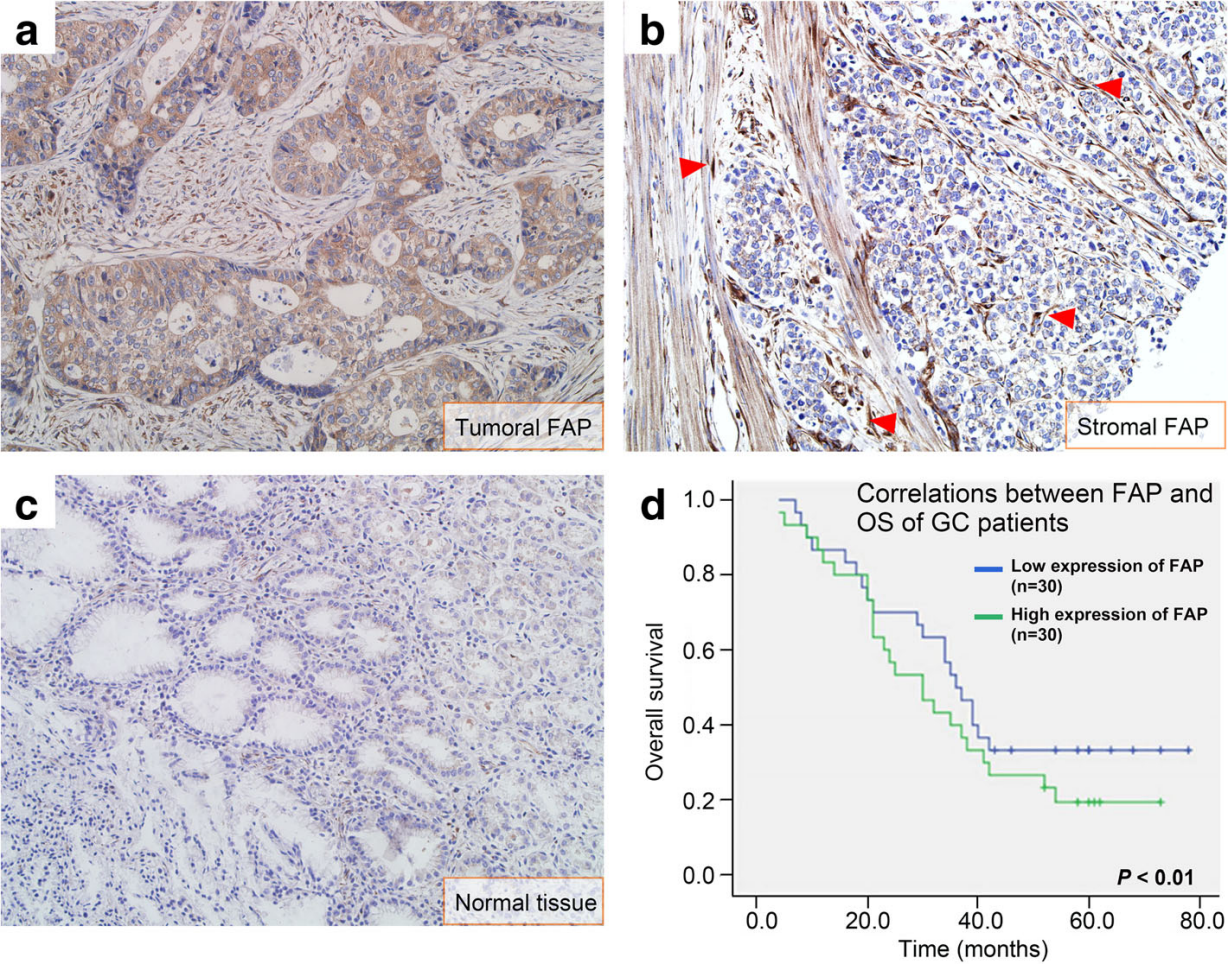
Correlations between FAP and OS of GC patients. FAP was expressed both in GC cells (**a**) and stromal CAFs (**b**, shown by red arrowheads). **c** FAP was not expressed in normal tissues. **d** The median OS of GC cases with high expression of FAP (30.2 months) was shorter than that with low expression of FAP (37.8 months), the difference was statically different (*P* < 0.01)

A total of 60 patients were included in this study, detailed information about patients’ demographics, clinicopathological characteristics was shown in Table 1. There were 4 groups including I (*n* = 12), II (*n* = 13), III (*n* = 27), and IV (*n* = 8). FAP was mainly expressed in cancer cells or CAFs (Fig. 1a, b). The positive ratio of FAP was 91.7% in GC tissues (*n* = 55). The FAP positive CAFs in GC tissues (32.80 ± 19.3) was much higher than that in peritumoral tissues (0.41 ± 0.21), the difference was statically different (*P* < 0.01).

**Table 1:** The relationship between stromal FAP and pathological characteristics in patients with gastric cancer

Variables	No. (%)	FAP positive CAFs	P*
Gender			0.309
Male	35 (62.5%)	34.8 ± 12.6	
Female	25 (37.5%)	30.7 ± 11.5	
Age (Means ± SD, yrs)			0.254
< 60	32 (55.0%)	38.9 ± 10.1	
≥ 60	28 (45.0%)	35.7 ± 13.2	
Tumor diameter			**0.024**
< 5 cm	34 (60.0%)	28.2 ± 15.2	
≥ 5 cm	26 (40.0%)	42.8 ± 20.4	
Differentiation degrees			**0.002**
Poorly-differentiated	28 (45.0%)	45.4 ± 13.0	
Moderately-differentiated	17 (30.0%)	35.6 ± 15.5	
Well-differentiated	15 (25.0%)	16.3 ± 8.6	
TNM stage			**0.001**
Stage I/II	25 (42.5%)	24.5 ± 6.4	
Stage III/IV	35 (57.5%)	57.1 ± 20.1	

*P-value in bold indicates the difference was statically significant

FAP expression correlated with the tumor diameter (*P* = 0.024), tumor differentiation degrees (*P* = 0.002), and TNM stage (*P* = 0.001), but not correlated with age and gender (*P* > 0.05 for all). According to the median value of FAP positive CAFs, GC cases were divided into high expression of FAP group (*n* = 30) and low expression of FAP group (n = 30). The median OS of GC cases with high expression of FAP (30.2 months) was shorter than that with low expression of FAP (37.8 months), the difference was statically different (*P* < 0.01, Fig. 1c).

### The construction of HELF^FAP^ cells with overexpression of FAP

The infection efficiency of FAP-copGFP was 100% at 72 h after infection and puromycin-based screening, which was indicated by green fluorescence. FAP expression was significantly elevated in HELF^FAP^ cells by nearly sixtyfold than HELF^NC^ cells. The IF results also indicate significantly higher FAP protein expression within HELF^FAP^ cells (Additional file 2: Figure S1). Therefore, HELF^FAP^ cells with overexpression of FAP were constructed for further studies, including cell proliferation, migration, invasion, as well as apoptosis.

### Stromal FAP promotes the proliferation, migration, and invasion abilities of SGC7901

**Fig. 2:**
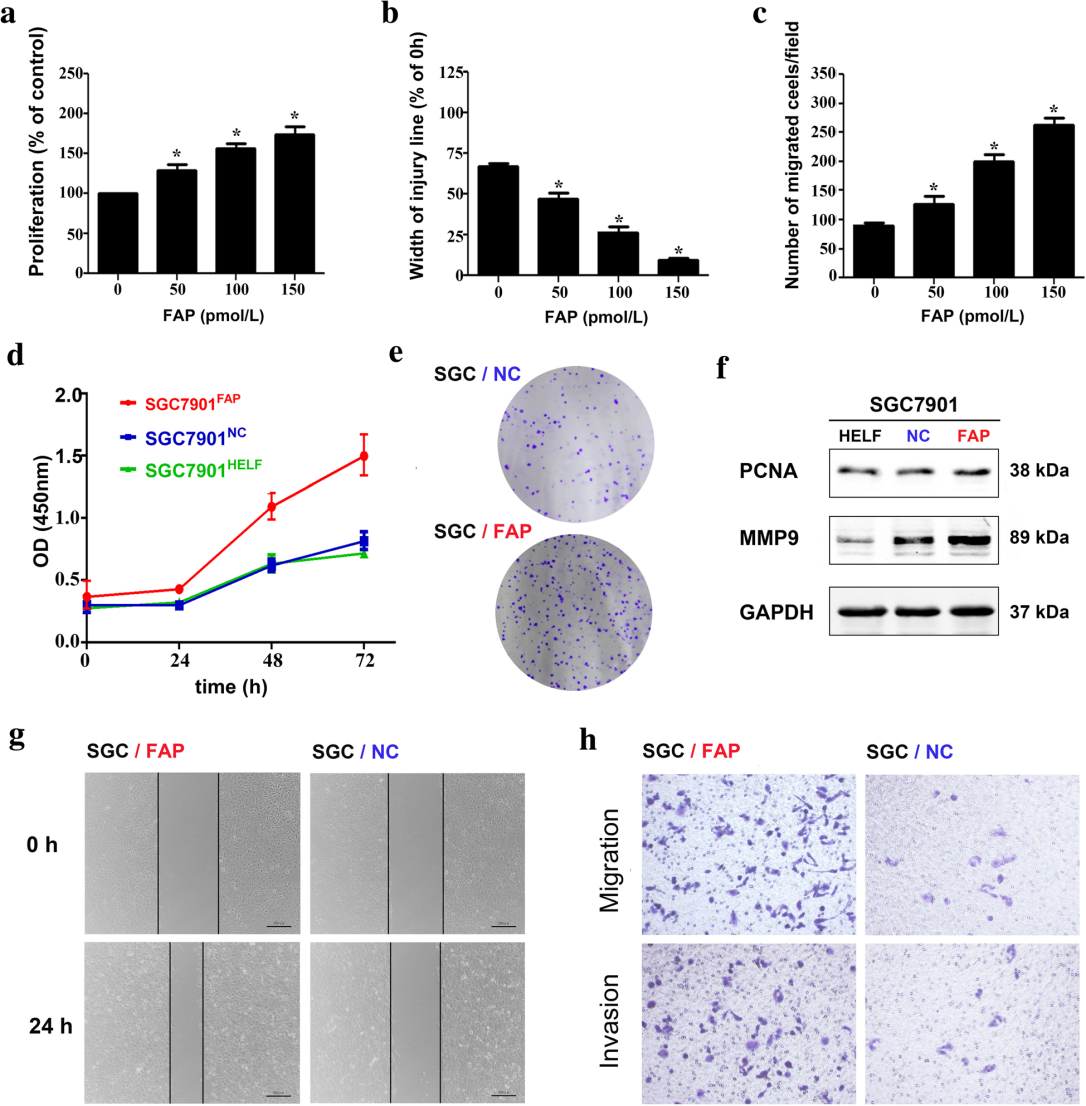
Stromal FAP promotes the proliferation, migration, and invasion abilities of SGC7901. In advance, SGC-7901 cells had been cocultured with HELF (SH group), HELF^NC^ cells (SN group) and HELF^FAP^ cells (SF group) for 72 h, respectively. **a** Exogenous FAP promotes SGC7901 proliferation in dose-dependent manner. **b** Exogenous FAP promotes SGC7901 migration in dose-dependent manner. **c** Exogenous FAP promotes SGC7901 invasion in dose-dependent manner. **d** Cell viability was determined by CCK8 assay. SGC7901 cells in SF group were promoted to proliferate. **e** Clone formation assay of SGC7901 cells in SF and SN groups. **f** Western blot assay indicated that the expression of PCNA and MMP9 in SF group was highest. **g** Wound healing assay indicated that the width of injury was lower in SF group in 24 h. **h** Cell migration and invasion abilities were determined by Transwell assay. The number of migrated and invasive SGC7901 cells in SF group was much higher than that in SN group

The proliferation and migration abilities of SGC7901 were significantly elevated by exogenous FAP in dose-dependent manner, as shown in Fig. 2a-c. The co-culture system went a further step to confirm this phenomenon. After co-cultured with HELF^FAP^, HELF^NC^ and HELF cells for 72 h, SGC7901 cells were harvested for CCK8 assays. The OD (450) value was recorded every 24 h to draw the proliferation curve, which indicated that the OD value of SGC7901^FAP^ was much higher (Fig. 2d). The number of SGC7901^FAP^ cells colony was also much higher than that of SGC7901^NC^ (Fig. 2e). Western blot assay indicated that the expression of PCNA and MMP9 protein in SGC7901^FAP^ were highest (Fig. 2f). The width of injury was lower in SGC7901^FAP^ cells in 24 h by wound healing assay (Fig. 2g). Cell migration and invasion abilities were determined by Transwell assay. The number of migrated and invasive SGC7901 cells in FAP group was much higher than that in NC group (Fig. 2h).

### Stromal FAP inhibits the apoptosis of SGC7901 cells

**Fig. 3:**
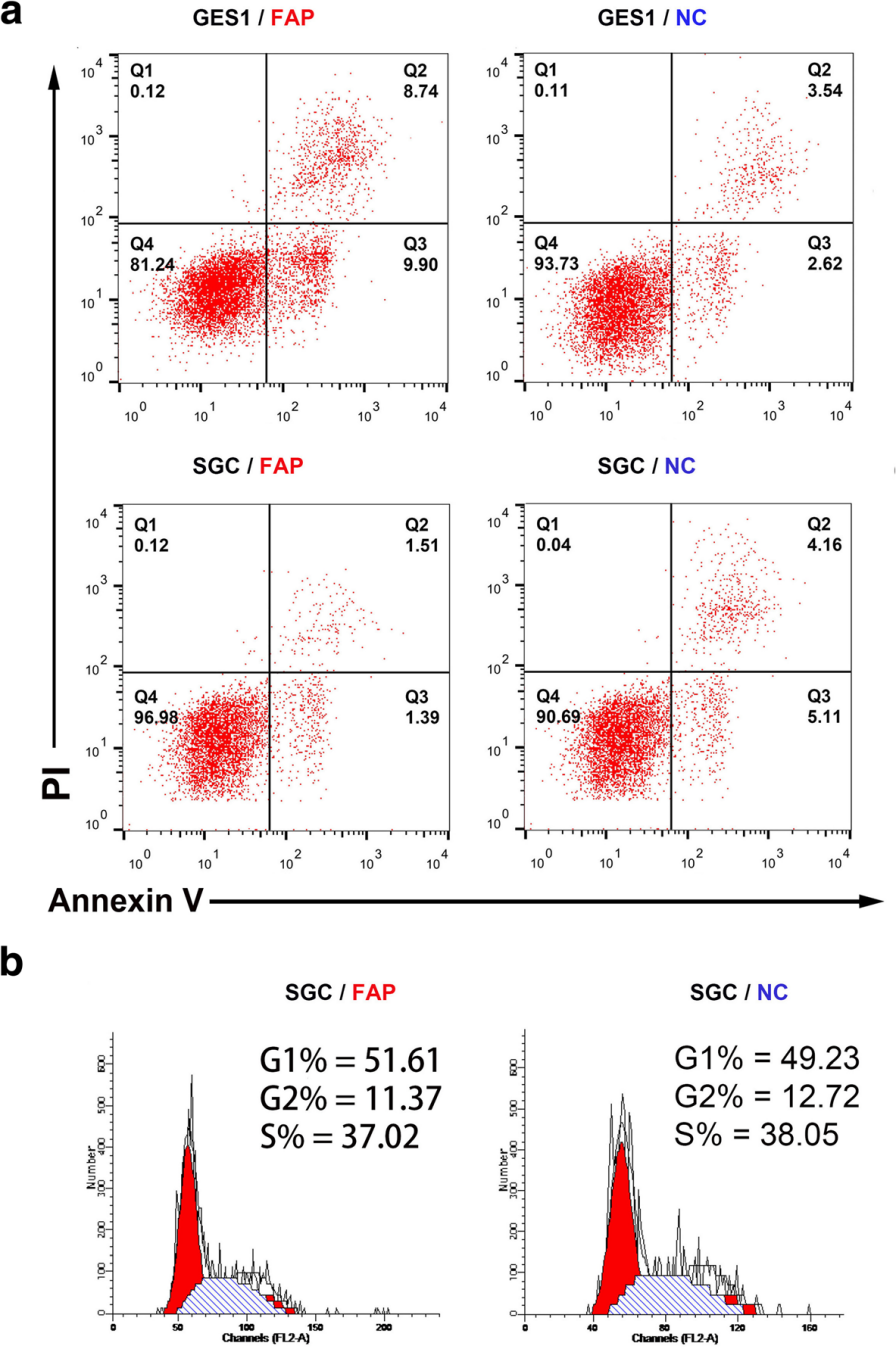
FAP inhibits the apoptosis of SGC7901 cells. **a** Apoptotic SGC7901 cells in NC group was higher than that in FAP group, whereas the result turned to the opposite in GES1 cells. **b** The cellular circle of SGC7901 was also detected by FCM. No differences could be observed in FAP and NC groups

The cocultured GES1 and SGC7901 cells were treated with cis-platinum meanwhile, then the apoptosis effect was detected by flow cytometry (FCM). Apoptotic SGC7901 cells in NC group was higher than that in FAP group, whereas the result turned to the opposite in GES1 cells (Fig. 3a). The cellular circle of SGC7901 was also detected by FCM to evaluate the potential reasons of apoptosis. However, no differences could be observed between FAP and NC groups (Fig. 3b). Then we hypothesized the potential correlation between FAP and Caspase family considering the apoptosis effect, whereas Western blot assay indicated that no significant differences regarding the expression of Caspase3, Caspase 9, Bax and Bcl-2 between FAP and NC groups (Additional file 3: Figure S2).

### Stromal FAP promotes EMT of SGC7901 through Wnt/β-catenin pathway

**Fig. 4:**
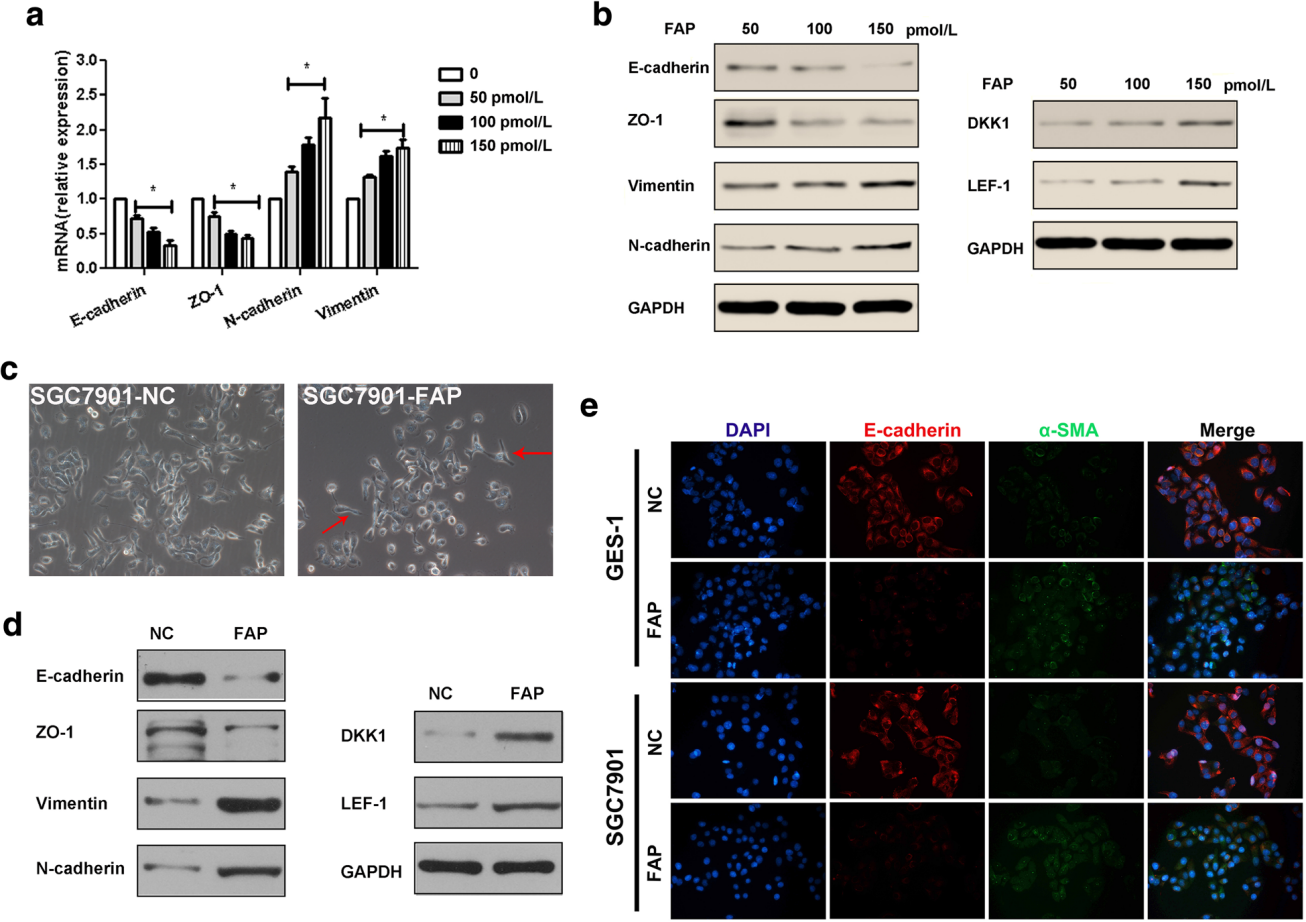
FAP promotes EMT of GC cells through Wnt/β-catenin pathway. Exogenous FAP promotes SGC EMT in dose-dependent manner. The expression of E-cadherin and ZO-1 were reduced, while that of N-cadherin and Vimentin were increased by qRT-PCR assay (**a**), and Western blotting assay (**b**). In addition, the DKK1 and LEF-1 protein, which could be participated in Wnt/β-catenin pathway, were also increased with more exogenous FAP. The result also accompanied in SGC cells co-cultured in SN and SF groups. **c** The morphology of SGC cells in SF group tended to be fibroblast-like, long fusiform, which was indicated by red arrows. **d** The expression of E-cadherin and ZO-1 were reduced, while that of N-cadherin and Vimentin were increased in SGC cells of SF group by Western blotting assay. Similarly, the DKK1 and LEF-1 protein were also increased. **e** The expression of E-cadherin was reduced, while that of α-SMA was increased both in GES1 cells (gastric normal cells) and SGC cells of SF group by the immunofluorescence staining

Exogenous FAP promotes EMT in dose-dependent manner. The expression of E-cadherin and ZO-1 were reduced, while that of N-cadherin and Vimentin were increased by qRT-PCR assay (Fig. 4a), and Western blotting assay (Fig. 4b). In addition, the DKK1 and LEF-1 protein, which could be participated in Wnt/ β-catenin pathway, were also increased with more exogenous FAP. The result also accompanied in SGC7901 cells co-cultured in NC and FAP groups.

The morphology of SGC7901 cells in FAP group tended to be fibroblast-like, long fusiform, which was indicated by red arrows in Fig. 4c. The expression of E-cadherin and ZO-1 were reduced, while that of N-cadherin and Vimentin were increased in SGC cells of SF group by Western blotting assay. Similarly, the DKK1 and LEF-1 protein were also increased (Fig. 4d). The expression of E-cadherin was reduced, while that of α-SMA was increased both in GES1 cells and SGC7901 cells of FAP group by the IF staining (Fig. 4e).

### Stromal FAP promotes GC progression in a xenograft gastric cancer nude mouse model

**Fig. 5:**
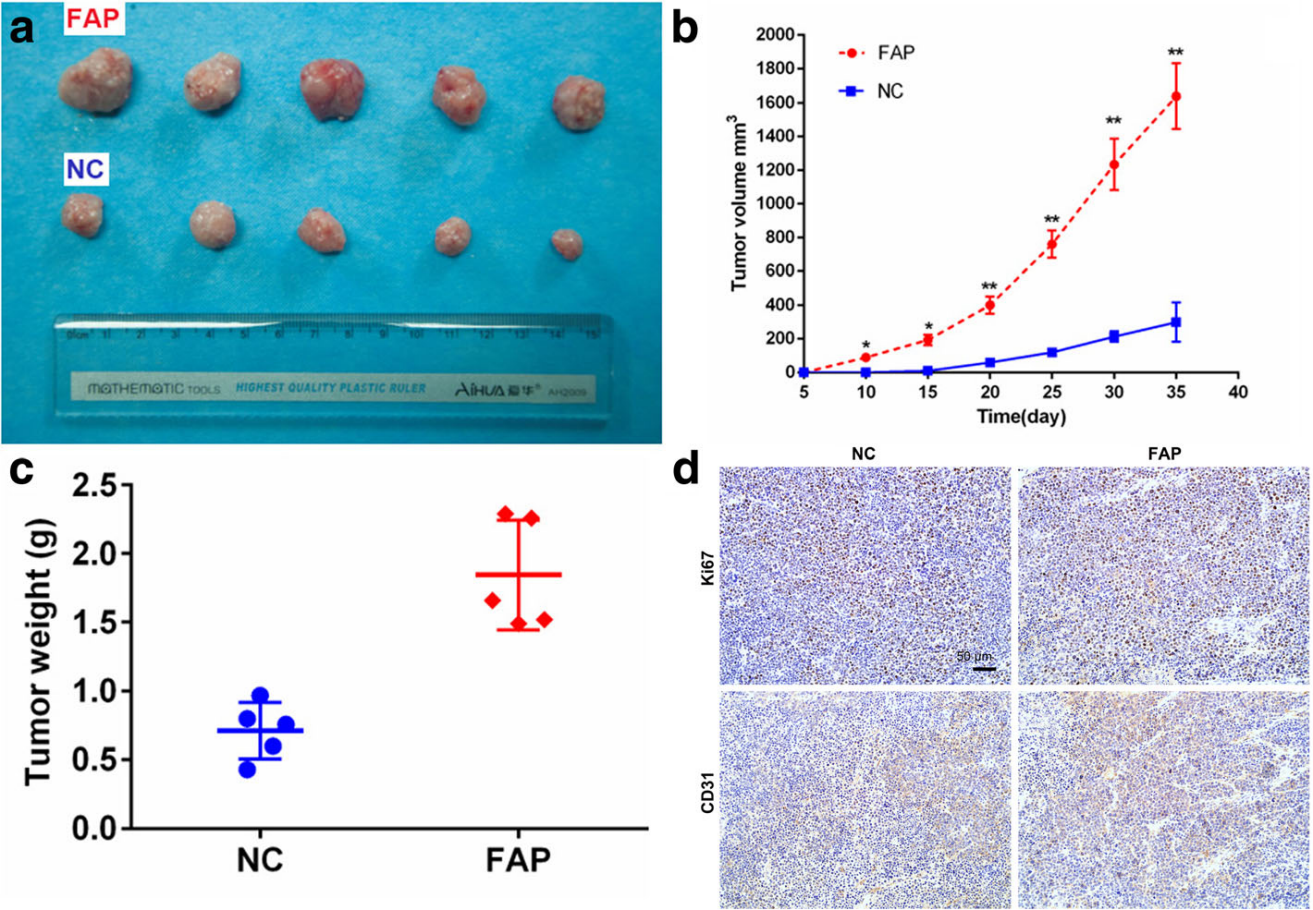
FAP promotes GC progression in a xenograft gastric cancer nude mouse model. **a** The combination of SGC7901 and HELF^FAP^ was much more effective in elevating tumor burden. The tumor volume (**b**) and weight (**c**) in the NC group were significantly lower than FAP group. **d** Representative immunohistochemical analysis of CD31, Ki67 (200× magnifications, Scale bar 50 μm)

To investigate the in vivo effects of stromal FAP, we examined the tumor promoting effect of FAP in a xenograft gastric cancer nude mouse model. SGC7901 cells (3 × 10^6^) were implanted subcutaneously in the right flank of nude mice, accompanied with HELF^FAP^ (1 × 10^6^) (*n* = 5) and HELF^NC^ (1 × 10^6^) (n = 5) cells, respectively. The combination of SGC7901 and HELF^FAP^ was much more effective in elevating tumor burden (Fig. 5a). The tumor volume and weight in the NC group were significantly lower than FAP group (Fig. 5b, c). Ki67 and CD31were examined by immunohistochemistry in the tumor sections. Both Ki67 and CD31 expression were elevated in FAP group. Taken together, stromal FAP promotes GC progression in a xenograft gastric cancer nude mouse model.

## Discussion

In this study, stromal FAP levels correlated with adverse clinic-pathological characteristics in GC, including larger tumor diameter, poorly tumor differentiation degrees, and advanced TNM stage. Therefore, FAP overexpression might contribute to cancer progression. Similar results could be summarized in colorectal cancer [19], pancreatic adenocarcinoma [20] and esophageal malignancies [21]. Unlike previous studies, our work provided a new insight into stromal FAP derived from CAFs in microenvironment [14]. The number of FAP positive CAFs were used to stratify GC patients into low- and high-risk groups. Consequently, the median OS of high-risk group was shorter. Therefore, stromal FAP might be closely related to GC progression and a potential prognostic biomarker.

Further biochemical and animal studies were conducted to ascertain the role of FAP as a causative and mechanistic biomarker. Although previous studies illustrated that FAP could promote cancer cells proliferation and invasion in various malignancies, for instance HO-8910 PM ovarian cancer cells [22], the TME-derived causations were ignored. Momentum evidence had confirmed the predominant function of TME during cancer invasion and metastasis [23–25]. In fact, the tumor initiation and growth were partially depended on stromal CAFs [11, 26]. According to TME theory, a co-culture model and a xenograft nude mouse model were used to mimic the cross talk between CAFs and GC cells. Herein, the exogenous FAP and HELF^FAP^ cells were found to promote the proliferation, migration and invasion abilities of GC cells in vitro by a series of functional assays. Therefore, it went a step further to detect tumor promoting functions of stromal FAP.

Except for sustaining proliferative abilities, resisting cell death and apoptosis was also the hallmarks of cancer [27]. Herein, stromal FAP inhibited the GC apoptosis, but induced normal mucosa epithelium apoptosis. Hence stromal FAP might be tumorigenic by destroying gastric epithelial cells and sustaining GC malignancies. Then we could hypothesize that, like other stromal components [28], CAFs were remodeled to support GC progression. As known, the main effect of apoptosis was mediated by Caspase-3 [29] and Caspase-9 [30] activation. The pro-apoptosis protein Bax might also be involved in by releasing cytochrome c from mitochondria and caspase-dependent pathway [31]. In this study, no similar phenomena could be found, thereby making it necessary to further explore underlying mechanisms.

Accumulating evidence indicated that EMT was a complex and dynamic process utilized by cancer cells during invasion and metastasis [32]. Once EMT occurred, cells lose the cell polarity and cell-cell contact, and gain mesenchymal properties, for instance increased motility [33]. The inducers of EMT can downregulate E-cadherin and upregulate N-cadherin and vimentin through modulating EMT-related signaling pathways, for instance WNT/β-catenin [34]. Dkk1, an antagonist of Wnt/β-catenin signaling, partially reverses the expression of EMT-associated proteins [35], and inversely correlated with cells apoptosis [36]. Herein, we reported corresponding results of E-cadherin, ZO-1, N-cadherin, vimentin, DKK1, and LEF-1. As a result, the above discussed functional roles of stromal FAP could be induced by EMT through Wnt/β-catenin signaling.

## Conclusion

In summary, we went a step further to characterize the biological processes and potential mechanisms associated with stromal FAP overexpression in GC. Stromal FAP derived from CAFs could promote GC progression via EMT mechanism through Wnt/β-catenin pathway.

## Additional files

Additional file 1: **Table S1.** Primers sequences in this study. (DOCX 15 kb)

Additional file 2: **Figure S1.** The construction and identification of HELF^FAP^ cells. (a) HELF^FAP^ cells in the bright field and the fluorescence field. The infection efficiency of FAP-copGFP was 100% at 72 h after infection. (b) The expression of FAP in HELF^FAP^ cells was significantly elevated in HELF^FAP^ cells by nearly sixtyfold through qRT-PCR assay, the difference was statically different (*P* < 0.001). (c) The immunofluorescence staining of FAP protein in both HELF^NC^ and HELF^FAP^ cells. FAP was overexpressed in HELF^FAP^ cells. (JPG 6016 kb)

Additional file 3: **Figure S2.** Western blot assay indicated that no significant differences were found regarding the expression of caspase3, caspase 9, Bax and Bcl-2 in SGC7901 cells between FAP and NC groups. (JPG 217 kb)

## Abbreviations

CAFs: cancer-associated fibroblasts
EMT: epithelial-mesenchymal transition
FAP: fibroblast activation protein
GC: gastric cancer
IHC: immunohistochemistry
OS: overall survival
PC: peritoneal carcinomatosis
TME: tumor microenvironment
