# Supplementary material for: Stromal fibroblast activation protein alpha promotes gastric cancer progression via epithelial-mesenchymal transition through Wnt/ β-catenin pathway
Source: BMC Cancer. 2018 Nov 12;18:1099. doi: 10.1186/s12885-018-5035-9 (PMC6233532; doi:10.1186/s12885-018-5035-9)
Supplement: Supplementary file 1 — Table S1. Primers sequences in this study. (DOCX 15 kb) [file 12885_2018_5035_MOESM1_ESM.docx]

**Supplementary Table 1** Primers sequences in this study

| ID | Primer sequences | |
| --- | --- | --- |
| FAP | Forward: | 5'-TGCGTATGTAGGTCCCCAGG-3' |
|  | Reverse: | 5'-CCATGTCTGCCAGTCTTCCC-3' |
| E-cadherin | Forward: | 5'-GCCCCATCAGGCCTCCGTTT-3' |
|  | Reverse: | 5'-ACCTTGCCTTCTTTGTCTTTGTTGGA-3' |
| ZO-1 | Forward: | 5'-CACGCAGTTACGAGCAAG-3' |
|  | Reverse: | 5'-TGAAGGTATCAGCGGAGG-3' |
| N-cadherin | Forward: | 5'-TGGACCATCACTCGGCTTA-3' |
|  | Reverse: | 5'-ACACTGGCAAACCTTCACG-3' |
| Vimentin | Forward: | 5'-CCTGAACCTGAGGGAAACTAA-3' |
|  | Reverse: | 5'-GCAGAAAGGCACTTGAAAGC-3' |
| GAPDH | Forward: | 5'- GGTCGGAGTCAACGGATTTG-3' |
|  | Reverse: | 5'- GGAAGATGGTGATGGGATTTC-3' |
