# Supplementary figures and images for: Stromal fibroblast activation protein alpha promotes gastric cancer progression via epithelial-mesenchymal transition through Wnt/ β-catenin pathway
Source: BMC Cancer. 2018 Nov 12;18:1099. doi: 10.1186/s12885-018-5035-9 (PMC6233532; doi:10.1186/s12885-018-5035-9)

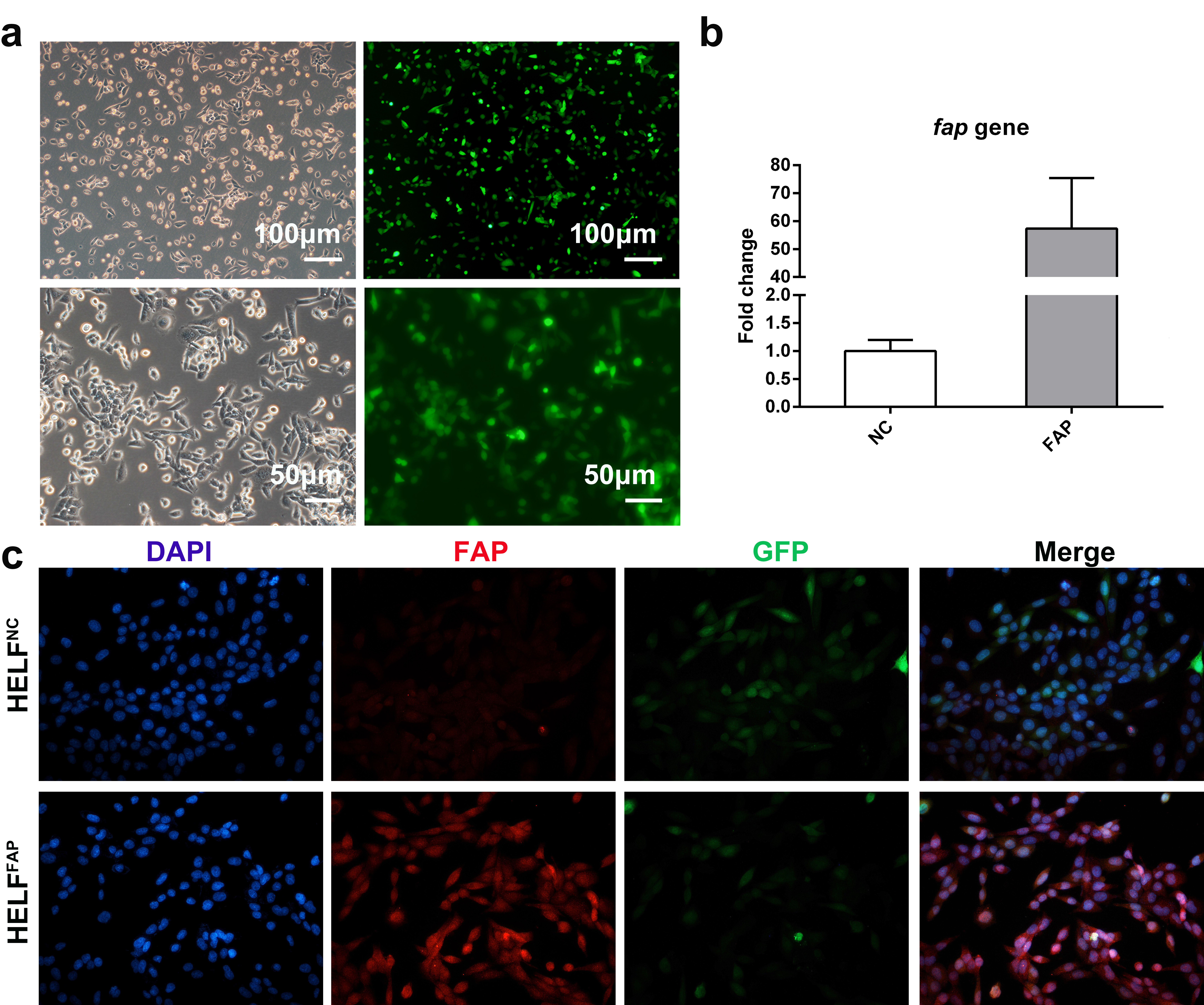

Supplement: Supplementary file 2 — Figure S1. The construction and identification of HELFFAP cells. (a) HELFFAP cells in the bright field and the fluorescence field. The infection efficiency of FAP-copGFP was 100% at 72 h after infection. (b) The expression of FAP in HELFFAP cells was significantly elevated in HELFFAP cells by nearly sixtyfold through qRT-PCR assay, the difference was statically different (P < 0.001). (c) The immunofluorescence staining of FAP protein in both HELFNC and HELFFAP cells. FAP was overexpressed in HELFFAP cells. (JPG 6016 kb) [file 12885_2018_5035_MOESM2_ESM.jpg]

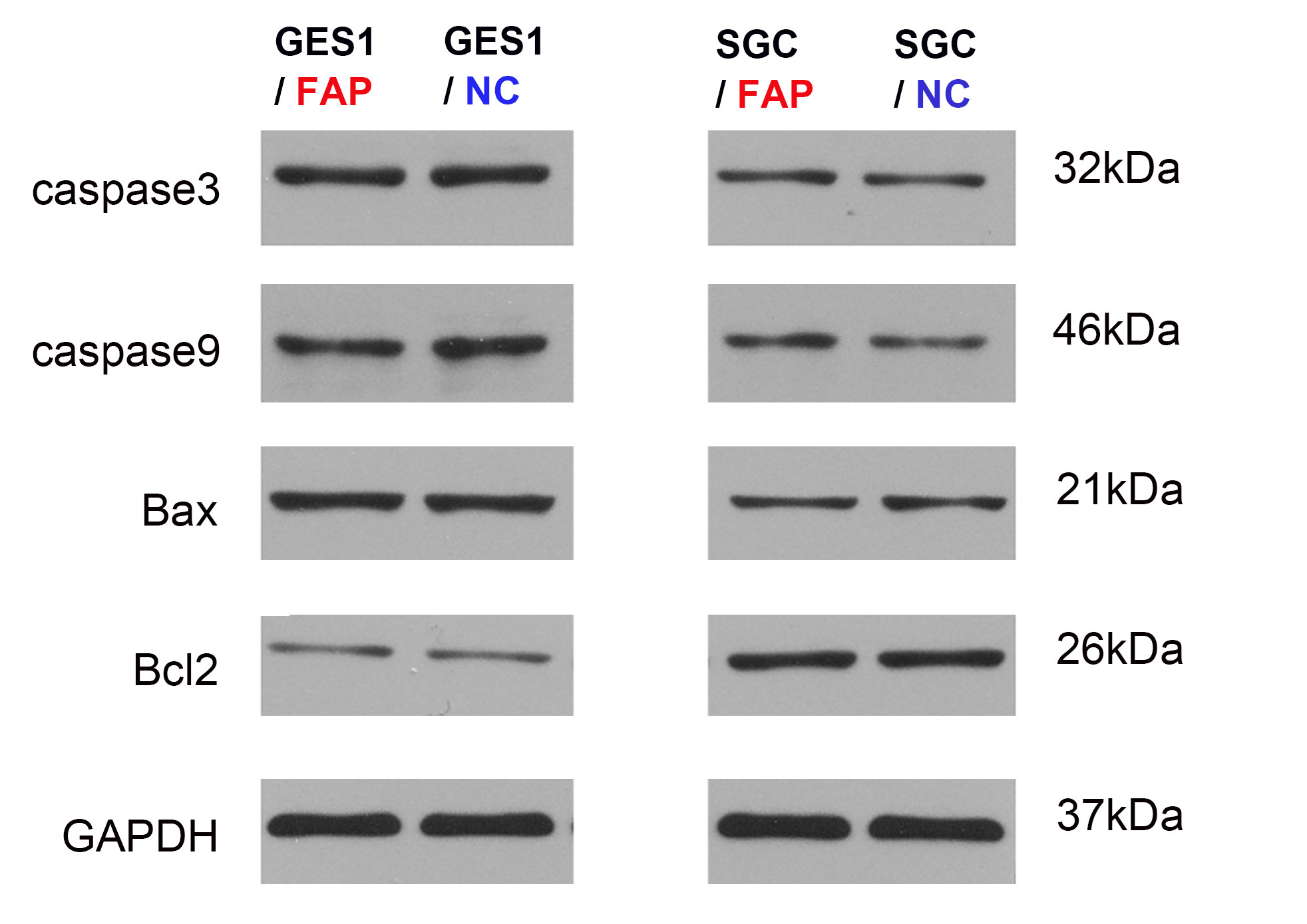

Supplement: Supplementary file 3 — Figure S2. Western blot assay indicated that no significant differences were found regarding the expression of caspase3, caspase 9, Bax and Bcl-2 in SGC7901 cells between FAP and NC groups. (JPG 217 kb) [file 12885_2018_5035_MOESM3_ESM.jpg]
